# Impact of Periosteal Branches and Septo-Cutaneous Perforators on Free Fibula Flap Outcome: A Retrospective Analysis of Computed Tomography Angiography Scans in Virtual Surgical Planning

**DOI:** 10.3389/fonc.2021.821851

**Published:** 2022-01-19

**Authors:** Michael Knitschke, Anna Katrin Baumgart, Christina Bäcker, Christian Adelung, Fritz Roller, Daniel Schmermund, Sebastian Böttger, Philipp Streckbein, Hans-Peter Howaldt, Sameh Attia

**Affiliations:** ^1^ Department of Oral and Maxillofacial Surgery, Justus-Liebig-University, Giessen, Germany; ^2^ Department of Diagnostic and Interventional Radiology and Pediatric Radiology, Justus-Liebig-University, Giessen, Germany

**Keywords:** virtual surgical planning, jaw reconstruction, CTA, flap failure, head and neck tumor, fibula free flap

## Abstract

**Background:**

Virtual surgical planning (VSP) for jaw reconstruction with free fibula flap (FFF) became a routine procedure and requires computed tomography angiography (CTA) for preoperative evaluation of the lower limbs vascular system and the bone. The aim of the study was to assess whether the distribution and density of periosteal branches (PB) and septo-cutaneous perforators (SCP) of the fibular artery have an impact on flap success.

**Method:**

This retrospective clinical study assessed preoperative CTA of the infra-popliteal vasculature and the small vessel system of 72 patients who underwent FFF surgery. Surgical outcome of flap transfer includes wound healing, subtotal, and total flap loss were matched with the segmental vascular supply.

**Result:**

A total of 72 patients (28 females, 38.9 %; 44 males, 61.1 %) fulfilled the study inclusion criteria. The mean age was 58.5 (± 15.3 years). Stenoses of the lower limbs’ vessel (n = 14) were mostly detected in the fibular artery (n = 11). Flap success was recorded in n = 59 (82.0%), partial flap failure in n = 4 (5.5%) and total flap loss in n = 9 (12.5%). The study found a mean number (± SD) of 2.53 ± 1.60 PBs and 1.39 ± 1.03 SCPs of the FA at the donor-site. The proximal FFF segment of poly-segmental jaw reconstruction showed a higher rate of PB per flap segment than in the distal segments. Based on the total number of prepared segments (n = 121), 46.7% (n = 7) of mono-, 40.4% (n = 21) of bi-, and 31.5 % (n = 17) of tri-segmental fibula flaps were at least supplied by one PB in the success group. Overall, this corresponds to 37.2% (45 out of 121) of all successful FFF. For total flap loss (n = 14), a relative number of 42.9% (n = 6) of distinct supplied segments was recorded. Wound healing disorder of the donor site was not statistically significant influenced by the detected rate of SCP.

**Conclusion:**

In general, a correlation between higher rates of PB and SCP and the flap success could not be statistically proved by the study sample. We conclude, that preoperative PB and SCP mapping based on routine CTA imaging is not suitable for prediction of flap outcome.

## Introduction

Taylor presented the free fibula flap (FFF) for the first time in 1975 (1), and Hidalgo employed it for mandible reconstruction 14 years later (2). This flap has a high success rate and is commonly used in reconstructive surgery (3). It allows the treatment of both bone and soft tissue defects with a single free flap from a single donor site (4). The FFF is the gold standard in mandibular reconstruction as it may be molded to a nearly ideal form of the missing jaw sections (5). Sufficient jaw reconstruction improves the quality of life (QoL) after ablative cancer surgery. After successful treatment, the overall QoL is comparable to that of the general population (6, 7). The osseous FFF permits for stable long-term prosthetic rehabilitation with dental implants with manageable donor-site complications (8–11). Computed tomography (CT) scans and DICOM data sets of the donor and recipient sites are required for virtual surgical planning (VSP) and the facilitation of custom-made, laser-melted, patient-specific titanium osteosynthesis plates (12, 13), which becomes widespread routine in many reconstructive centers (14). MRA was found as a reliable and non-invasive technique to identify anatomical variants and arterial stenoses (15, 16) without radiation in preoperative FFF planning (17). But CTA has been shown to be better than MRA for perforator mapping (18), as well as being more widely available, adequately accurate, and economic (19–21). The method of VSP was described by Eckardt and Swennen in 2005 for mandible reconstruction (22) and becomes more popular since than (23–27). The transfer from virtual planning to operating fields became accurate due to the possible because of the three-dimensionally designed and configured plate (28). Thank this planning method an exact and predictable uni- and poly-segmental bone restorations are possible (14, 29, 30). Success rates of the FFF ranging between 90% to 95% have been reported in the literature (31–34). Despite these significant benefits, surgery remains challenging in terms of insufficient perforator vessels, vascular bundle complications, or inadequate resections margins (35, 36). A thorough preoperative examination of the vascular system using a computed tomography angiography scan (CTA) to reduce those risks is required, as CTA scans allow for simultaneous evaluation of bony and vascularly structures (37).

The descriptive term periosteal branch (PB) is very general and has to be precise. Studies showed that bone perfusion of the skeleton is maintained by a system of three types of vessels (38, 39): endosteal nutrient vessels, penetrating periosteal vessels, and non-penetrating periosteal vessels. There are crosslinks between periosteal and endosteal vessels but without clear borders of perfusion. Experimental studies show that the inner two-thirds of the cortical bone is supplied by the endosteal system and the outer third by the periosteal system (40). Age seems to play a vital role, as the endosteal supply dominates the perfusion of cortical bone in youth, while in advanced age, a greater cortical thickness can be supplied by periosteum (41). While the nutrient vessels contribute to periosteal and endosteal blood supply (41), the non-penetrating branches do not appear to have a contribution to the endosteal perfusion (39, 42). The FFF is supplied by the non-penetrating perforator vessel subtypes direct periosteal and musculo-periosteal and nutrient vessels (1, 43). Several studies supported the thesis that non-penetrating branches only perfuse the outer section of the cortical bone (42, 44).

An anatomical examination of 30 formalin-fixed legs revealed that 27 legs (90%) had a singular nutrient vessel, and two (6.6 %) had a double nutrient vessel. In one leg, no nutrient vessel was observed. These vessels enter the fibula predominantly in the middle third, at its medial crest. In contrast, only one entered from the posterior surface and showed, on average, a diameter of 0.9 mm – 1.5 mm (45). Based on 54 cadaveric legs, it was found that the fibular nutrient artery, which arose from the fibular artery as a short descending branch, penetrated the M. flexor hallucis longus to enter the fibular nutrient foramen (46). Between the distal half of the first-quarter and second-quarter segments of the fibula, the fibular nutrient artery, and up to three arcuate arteries were located constantly (47). The term periosteal branch summarizes, therefore, nutrient and non-penetrating vessels.

Previous radiological analyzes of our research group on the same study sample revealed different distribution patterns and frequencies for PB and SCP based on CTA scans of both legs. A bimodal distribution pattern for PB and three peaks for SCP in performed CTA for VSP were recorded (48). Further, significant differences concerning the number of periosteal branches in the bone segment of different sizes were found compared to cadaver studies (49). The more proximal the FFF segment, the more frequently a potential PB was observed in the CTA scans. So that a comparison of the previous published radiological findings to the clinical data of the same patient’s collection is of great interest, which is the topic of this paper.

This investigation aimed to evaluate the impact of detected small vessels (PB and SCP) on the surgical outcome after VSP of uni- and poly-segmental mandible reconstruction with FFF. Additionally, the following questions were evaluated in the study.

1. How do infra-popliteal branching pattern and fibular artery vascular anomalies (stenoses) affect the outcome of flap surgery?
2. How does the distribution of CTA-based detected PB and SCP influence the surgical result of mono- and poly-segmental jaw reconstructions with partial or total flap loss?
3. Does the observed distribution of PB and SCP impact wound healing of the donor site?

## Material and Methods

### Patient Collection, Ethical Consideration and Inclusion Criteria

The ethics committee of the Justus-Liebig-University Giessen approved the study (approval number: AZ33/20, approval date: 25.5.2020). No written obtained consent was required from the considered patients. Individuals meeting the following criteria were included: Immediate or delayed mandible reconstruction using FFF planned virtually, availability of preoperative CTA scans with a maximum slice thickness of 1.5 mm, treatment performed between January 2015 and December 2020.

**Figure 1:**
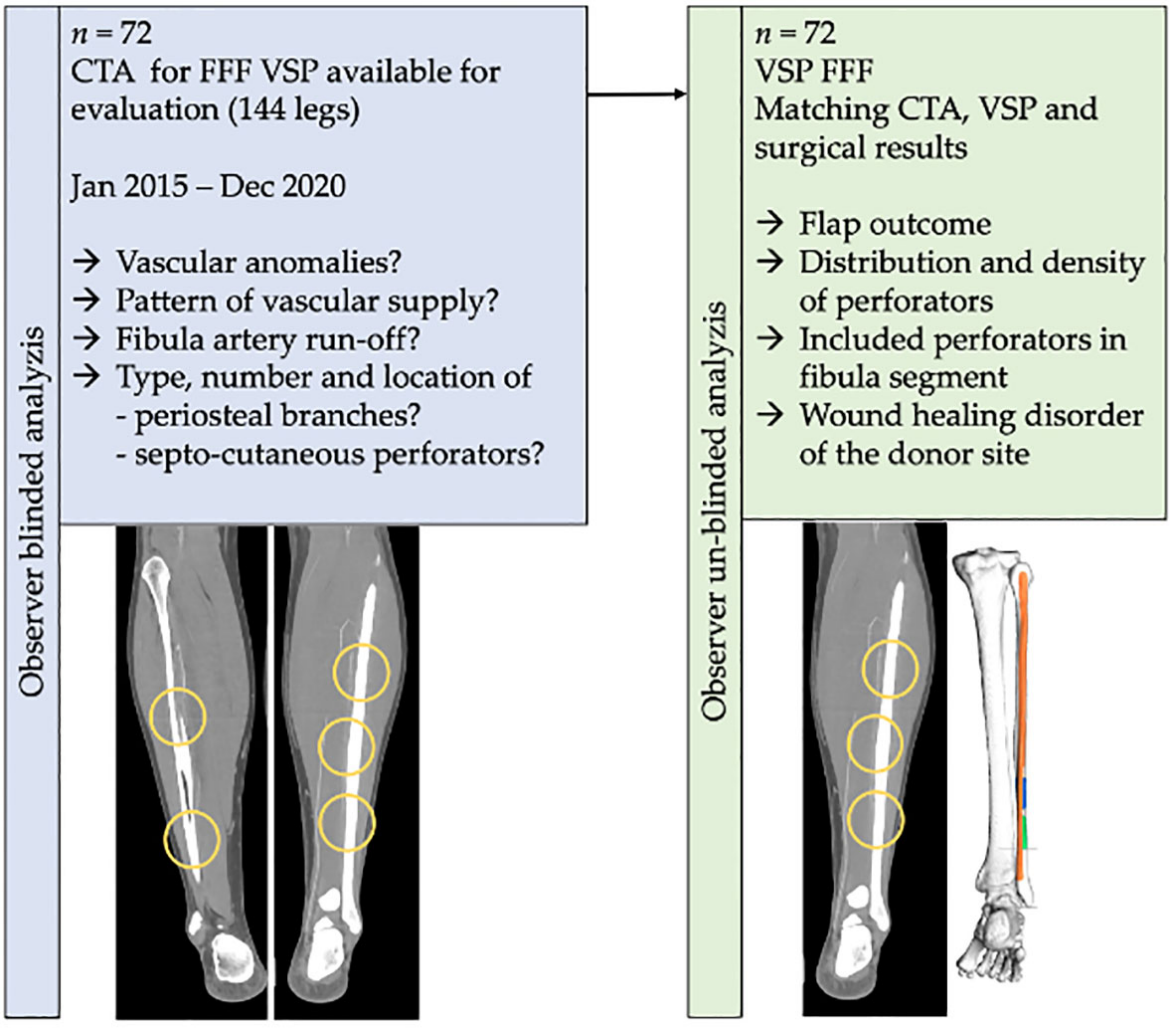
Workflow of the present study. A total number of n = 72 computed tomography angiography (CTA) DICOM-datasets of virtual planned jaw reconstructions with a free fibula flap were included in the investigation. Findings on the vascular infra-popliteal branching pattern, stenoses, and distribution and density of periosteal branches and septo-cutaneous perforators of the fibular artery were matched with flap surgery outcome.

A total number of 77 patients fulfilled the inclusion criteria. Five could not be included because of one fibula CTA after reconstruction with contralateral fibula after flap loss (n = 4) and after tumor recurrence (n = 1). Finally, 72 patients with CTA scans of 144 legs were available for the analyzis (**Figure 1**).

**Figure 2:**
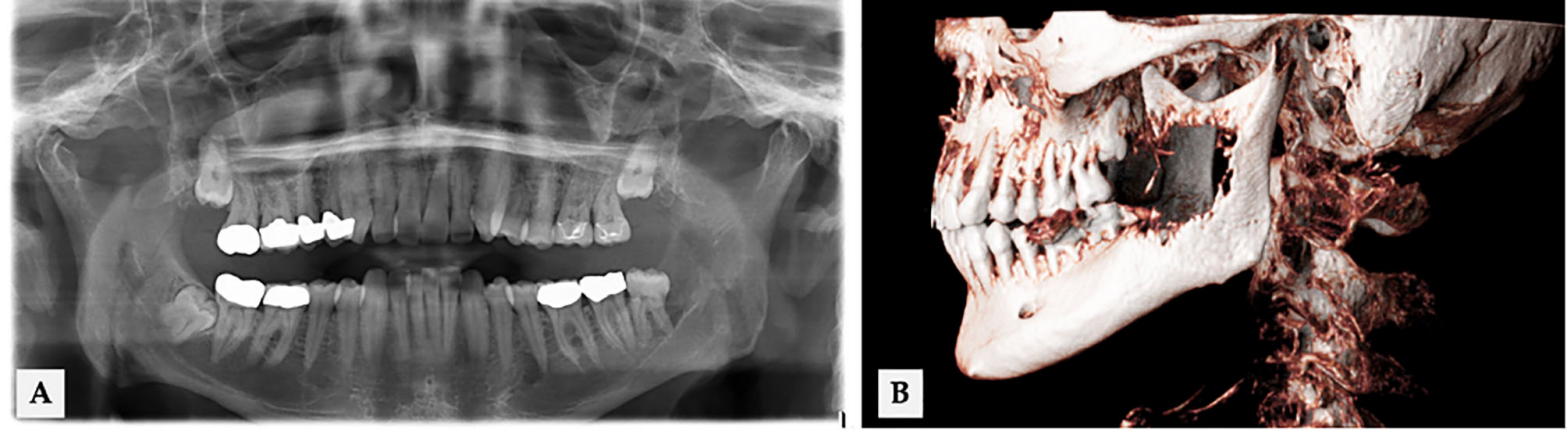
56 years old male with an infiltrative growth of oral squamous cell carcinoma (T4) in regio 38 (ID 18 in **Figure 5**). **(A)** Extension of osseous destruction in OPT and **(B)** cinematic volume rendering CT reconstruction.

**Figure 3:**
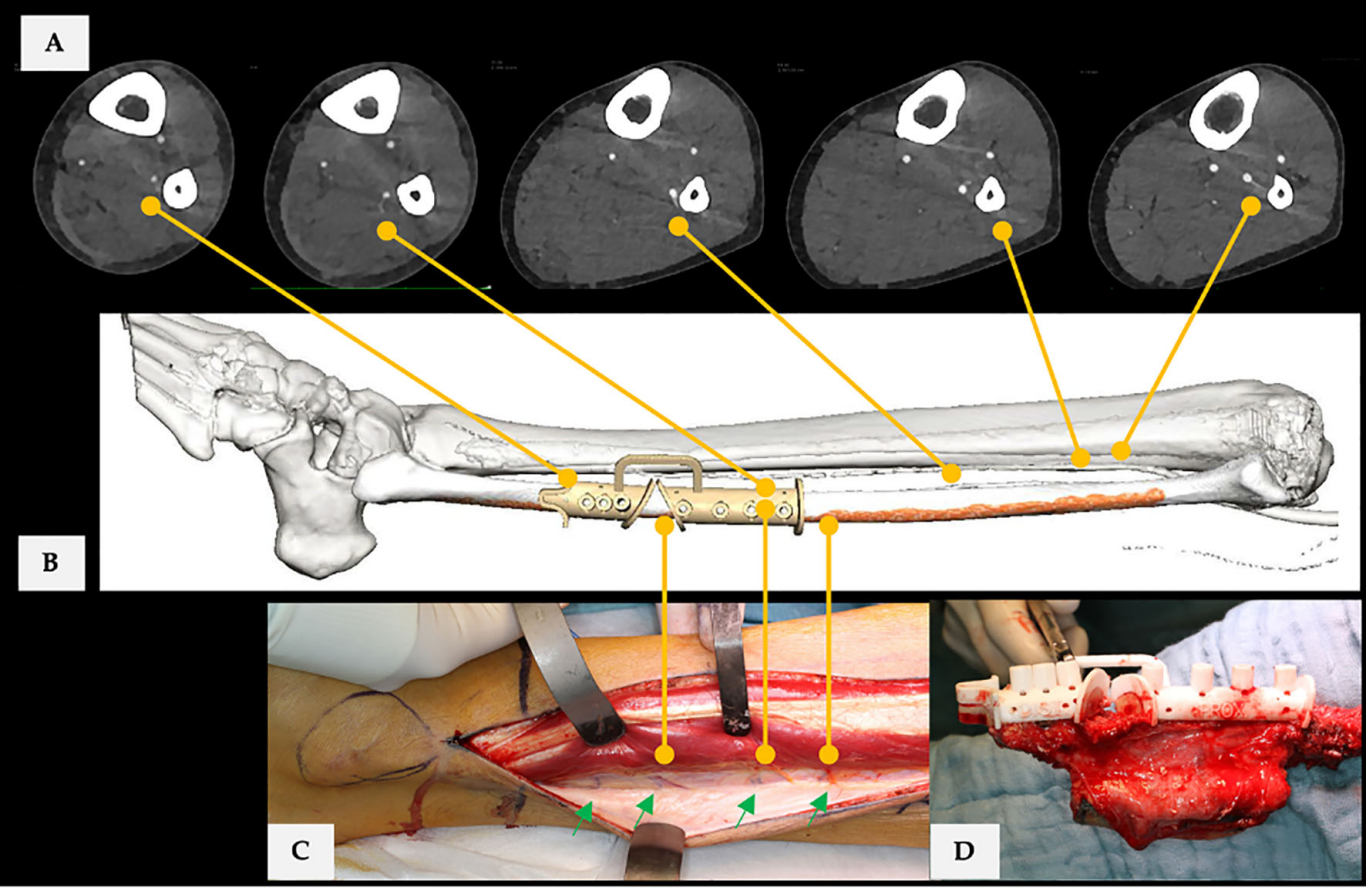
**(A)** CTA scan (axial plane) of the donor site. Yellow lines connect found PB and SCP with virtual surgical planning. **(B)** Final virtual surgical planning for bi-segmental mandible reconstruction with free fibula flap. **(C)** Yellow lines connect corresponding vessels with the operating field. **(D)** Applied cutting guide, performed osteotomies, and shaped neo-condyle. Case is ID 18 in **Figure 5**. In CTA assessment were 5 PB and one SCP of the FA at donor site recorded. Each fibula flap segment was supplied by one PB, while three were located proximal to the designed flap. The CTA-based SCP position was in the middle of the skin paddle in the proximal fibula flap segment. The radiological examination of the FA was without pathological findings (type I-B: infra-popliteal branching pattern, which means trifurcation of the popliteal artery in ATA, PTA, and FA). Overall, there were no radiological reservations or restrictions to surgery. **Figure 3A, C** shows that there was a discrepancy between the radiological and the operative findings. The number of SCP was at least 4 (green arrows).

**Figure 4:**
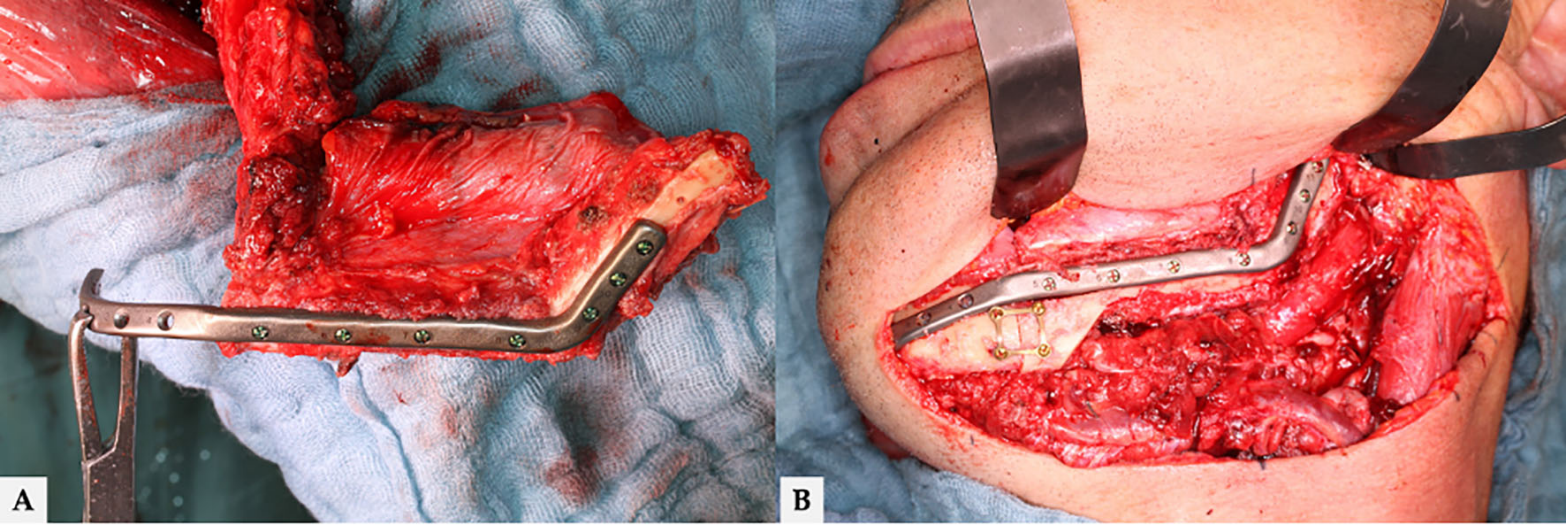
**(A)** Final molded bi-segmental composite fibula flap and **(B)** neo-mandible. Additional triangular free bone transplant to smooth the contour of neo-mandible’s jawline. Finally, total flap loss occurred in this case. The surgical revision revealed a combined arterial and venous thrombosis.

Dissection of the fibula flap was conducted using Gilbert’s lateral approach (50). A segment of 8cm at the proximal end and the distal end, a 6-8cm length, was left in place to preserve knee and ankle stability. When a composite flap was harvested, the perforators were protected with a muscle cuff of M. soleus and M. flexor hallucis longus. A summarized clinical example is given in **Figures 2**–**4**. Wound closure of the donor site was done primarily in cases of non-composite FFF. When composite FFF were harvested, all donor site defects were covered with meshed split thickness skin graft.

All CTA scans were done at the University Hospital Giessen’s Departments of Diagnostic and Interventional Radiology and Pediatric Radiology. The CT scans were done using a first-generation dual-energy CT scanner and a third-generation dual-energy CT scanner (SOMATOM Definition AS & Force, Siemens Healthineers, Forchheim, Germany). Above the aortic bifurcation to the feet, scans of both legs were performed with a slice thickness of 1.5 mm (70 kV, 300 mA max, pitch 0.5, collimation 0.6 mm, matrix size 512 x 512). Intravenously, non-ionic contrast fluids containing 350 mg of iodine per milliliter (Ultravist 370, Bayer, Leverkusen, Germany) were given. The amount of contrast media used is determined on the patient’s weight.

CTA DICOM data sets were analyzed in HOROS-Software for Mac (Version 4.0.0 RC5, Horosproject). Horos is a free and open-source code software (FOSS) program distributed free of charge under the LGPL license at Horosproject.org and sponsored by Nimble Co LLC d/b/a Purview in Annapolis, MD, USA. The CTA quality was assessed by side-by-side comparison with an ROI in the center of the popliteal artery and dorsal vessels of the dorsum of the foot. For every CTA, the measurements were performed on both patients’ legs.

### Study Parameters

The following parameters were collected in a previous investigation on the study sample: Length of the fibula, bone and vascular anomalies, vascular anatomy and branching pattern of the infra-popliteal vessels will the classified concerning Kim et al. (51), length of TTF, number and localization of SCPs and PBs from the distal tip of the fibula bone to branching and between the branches.

These findings were matched with the surgical outcome including: Patient’s age (at CT scan), gender, body height, and weight, BMI, flap-type (composite or non-composite flap), site of flap harvesting, distance to the distal tip of the fibula (ankle), as well as the number, length, and position of fibula segments. Additionally, total transplant length, which were taken out of the virtual planning report was recorded. Information about flap status (complete flap success, partial (bone or skin paddle), or total flap loss) was extracted from the medical records. PFF was defined as any loss of parts of the skin paddle (skin), parts or segments (poly-segmental reconstruction) of bone grafts (bone), or a combination of both (52). The donor site’s wound healing disorder (WHD) was classified as minor WHD when only a conservative wound had been performed. Major WHD implicates large wounds with exposed tendons and surgical treatment by applying split-thickness skin graft after wound debridement.

### Statistical Analysis

Pearson’s χ2 test, Fisher’s exact test, and the Freeman−Halton extension (53) were conducted on the categorical variables used to analyze flap outcome concerning: gender, flap-type (composite or non-composite flap), donor site, number of bone segments, and number and type of included perforators and ASA-score. Kruskal-Wallis test was performed to analyze defined flap outcome groups concerning metric parameters. The continuous parameters: age, body height, and weight, body-mass-index (BMI), the total length of the fibula, the length of the fibular artery (FA) from origin to the distal tip of the fibula bone, the diameter of the fibular artery, the length and the diameter of the truncus tibiofibularis (TTF), the number and the distance of septo-cutaneous perforators (SCP), the periosteal branches (PB), overall reconstruction length, and the segment length were verified for normality. The distribution was presented as a mean (standard deviation), and Student’s t-test was performed. p < 0.05 was defined as statistically significant. The statistical analyzes were carried out with SPSS (IBM SPSS Statistics, v28.0, Armonk, NY, USA).

## Results

A total of 72 patients (28 women, 38.9 %; 44 men, 61.1 %) fulfilled the inclusion criteria. The mean age was 58.5 ± 15.3 years (range: 14.8 – 82.6 years). Firstly, the vascular system of the study sample was assessed and the sample was categorized into donor and non-donor site for further analyzis.

In the gender-mixed sample, no significant difference in fibular bone length was found. Concerning the infra-popliteal branching pattern type as classified by Kim et al., all donor fibulae had a regular vascular supply equivalent to types I-A through II-C. In contrast, at the non-donor site, two cases of type III-A and two cases of type III-B were found (51). Out of 144 legs, 88.9 % (n = 128) were assigned as type I-A. Detailed evaluation of the donor site vascular architecture revealed that type I-A was found in 93.1 % (non-donor site: 84.7 %). Two donor site vascular systems were classified as type I-B, and one case was assigned to categories I-C to II-B. Four legs of the non-donor site showed dominant fibular artery (FA) variants (III-A: n = 2; III-B: n = 2). No type III-C branching pattern was observed, defined as a dominant fibular artery, that can lead to critical perfusion (**Tables 1** and **2**).

**Table 1:** CTA assessment for fibular bone and vascular system parameters of the study sample.

*n* = 144	Donor site (*n* = 72)	Non-donor site (*n* = 72)	Total	*p-*value
Fibula length, mean (mm) ± SD	373.9 ± 30.2	372.8 ± 30.9	142	0.829
Fibula bone anomalies				
Fracture	0	1	1	–
Branching pattern of the calf (51)				
Regular (I-A to II-C)	72	68	140	
Absent ATA (III-A)	0	2	2	
Absent PTA (III-B)	0	2	2	0.119
Stenoses				
ATA	0	1	1	
PTA	0	2	2	
FA	5	6	11	0.670
Length of TTF, mean (mm) ± SD	32.6 ± 12.9 (*n* = 67)	32.5 ± 14.6 (*n* = 61)	128	0.965
Diameter of TTF, mean (mm) ± SD	4.13 ± 0.95 (*n* = 67)	4.16 ± 1.0 (*n* = 61)	128	0.862
Length of FA, mean (mm) ± SD	244.9 ± 36.9	243.0 ± 43.3	142	0.777
Diameter of FA, mean (mm) ± SD	3.12 ± 0.79	3.21 ± 0.78	142	0.493
Overall found SCP, *n* (%)	101 (47.4%)	112 (52.6%)	213	
Diameter SCP, mean (mm) ± SD	0.93 ± 0.28	0.93 ± 0.32	0.93 ± 0.30	1.0
Mean SCP per fibula (mm) ± SD	1.39 ± 1.03	1.52 ± 1.23	1.40 ± 1.01	0.407
Overall found PB, *n* (%)	185 (51.2%)	176 (48.8%)	361	
Diameter PB, mean (mm) ± SD	0.87 ± 0.24	0.87 ± 0.26	0.87 ± 0.56	1.0
Mean PB per fibula (mm) ± SD	2.53 ± 1.60	2.42 ± 1.60	2.47 ± 1.54	0.514

SD, standard deviation; ATA, anterior tibial artery; FA, fibular artery; PB, periosteal branch; PTA, posterior tibial artery; SCP, septo-cutaneous perforator; TTF, truncus tibiofibularis.

**Table 2:** Infrapopliteal arterial branching variations were classified by Kim (51) of the investigated sample (n = 144).

Type	Donor site (*n* = 72)	Non-donor site (*n* = 72)	Total (*n* = 144)
	*n* (%)	*n* (%)	*n* (%)
I-A	67 (93.1)	61 (84.7)	128 (88.9)
I-B	2 (2.8)	1 (1.4)	3 (2.1)
I-C	1 (1.4)	–	1 (0.7)
II-A	1 (1.4)	2 (2.8)	3 (2.1)
II-B	1 (1.4)	4 (5.6)	5 (3.6)
II-C	–	–	–
III-A	–	2 (2.8)	2 (1.4)
III-B	–	2 (2.8)	2 (1.4)
III-C	–	–	–

At all, 14 stenoses of the lower limbs’ vessels were recognized. Five stenoses of the FA were detected at the donor site, while in the non-donor site, stenoses in all three vessels had been found (FA: n = 6; ATA: n = 1; PTA: n = 2). In donor vs. non-donor site comparison, no significant differences for the total length of the TTF and FA and the diameters were found. PB and SCP were located in equal parts at donor- vs. non-donor sites. The study detected a mean number (± SD) of 2.53 ± 1.60 PB and 1.39 ± 1.03 SCP of the FA at the donor-site in the region of interest between the exit of FA from the TTF and 5.0 cm above the distal tip of the fibula bone. Compared to the non-donor site, a non-significant difference in the mean number of recorded PB and SCP was found.

**Figure 5:**
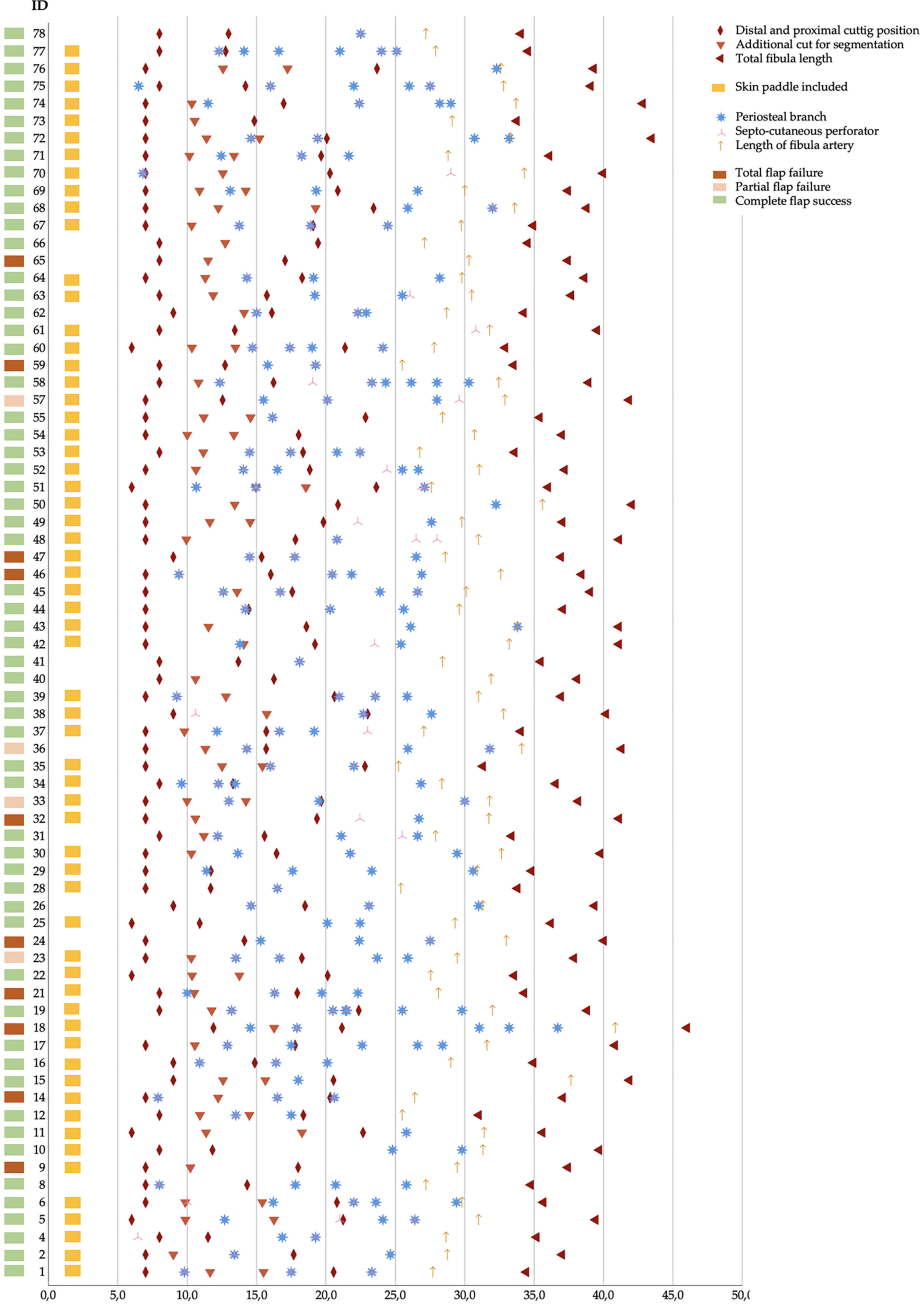
Sample of n = 72 virtual planned jaw reconstructions. Matching virtually planned parameters, CTA detected vessels (PB and SCP), and surgical outcome. All position marks (x-axis) are given in centimeters.

The findings were matched with virtual surgical planning (VSP) and surgery results, and flap outcome was categorized concerning complete flap success (FS), partial (PFF), and total flap failure (TFF). Partial flap failure was defined as (sub-)total loss of the skin paddle and/or parts or segments of poly-segmental reconstructions. The detailed results are summarized in **Table 3** and **Figure 5**. Total flap loss was recorded in n = 9 cases (12.5%). The highest average age with 64.9 ± 8.0 years was found in the TFF-group, while the lowest mean age with 49.8 ± 20.6 years was estimated in the PFF group. The finding was without statistical significance. Differences concerning body weight were found significant for PFF in comparison to FS (PFF: 92.3 ± 10.6 kg vs. TFF: 64.9 ± 8.0 kg; p = 0.012) and a trend towards significance concerning the TFF (PFF: 92.3 ± 10.6 kg vs. FS: 58.4 ± 15.6 kg; p = 0.061). About 43.1 % of the study sample were classified at least ASA-score 3. PFF and TFF were found only for ASA-score 2 and 3 and within each class in equal proportions. All registered PFFs and TFFs (except for one type, I-B) occurred in a I-A branching pattern.

**Table 3:** Demographic and surgery-associated parameters.

*n* = 72	Flap success	Partial flap failure	Total flap failure	*p*-value
	59 (82.0%)	4 (5.5%)	9 (12.5%)	
Age (years), mean ± SD	58.4 ± 15.6	49.8 ± 20.6	64.9 ± 8.0	0.338
Gender, n (%)				
Male	33 (44.1)	4 (100.0)	7 (77.8)	
Female	26 (55.9)	0	2 (22.2)	0.150
Body weight (kg), mean ± SD	71.7 ± 15.7	92.3 ± 10.6	74.6 ± 15.2	*0.012
Body height (cm), mean ± SD	169.9 ± 10.0	179.3 ± 3.9	176.8 ± 11.3	0.067
BMI (kg/m^2^), mean ± SD	24.7 ± 5.2	28.8 ± 3.6	23.6 ± 4.8	0.189
ASA-score, n (%)				
1	3 (5.1)	0	0	
2	31 (52.5)	2 (50.0)	5 (55.6)	
3	23 (39.0)	2 (50.0)	4 (44.4)	
4	2 (3.4)	0	0	0.973
Reconstruction site				
Maxilla	15 (25.4)	1 (25.0)	2 (22.2)	
Mandibula	44 (74.6)	3 (75.0)	7 (77.8)	1.0
FFF type, n (%)				
Composite flap	51 (86.5)	3 (75.0)	7 (77.8)	
Non-composite flap	8 (13.5)	1 (25.0)	2 (22.2)	0.573
Donor site, n (%)				
Left	22 (37.3)	0	4 (44.4)	
Right	37 (62.7)	4 (100.0)	5 (55.6)	0.384
Distance to the tip of the fibula (ankle), mean ± SD				
60 mm	6 (10.2)	0	0	
70 mm	31 (52.5)	4 (100.0)	5 (55.6)	
80 mm	17 (28.8)	0	2 (22.2)	
90 mm	5 (8.5)	0	1 (11.1)	
118.9 mm	0	0	1 (11.1)	0.175
Number of segments, n (%)				
1	15 (25.4)	1 (25.0)	4 (44.4)	
2	26 (44.1)	2 (50.0)	5 (55.6)	
3	18 (30.5)	1 (25.0)	0	0.351
Total transplant length (mm), mean ± SD (range)				
1	56.1 ± 15.3 (35.0 – 94.9)	55.0	68.1 ± 17.8 (47.3 – 90.2)	0.458
2	106.6 ± 21.5 (71.0 – 143.6)	99.8 ± 18.2 (86.9 – 112.6)	109.9 ± 18.7 (90.5 – 133.1)	0.804
3	142.3 ± 21.2 (103.7 – 176.3)	126.7	–	0.361
Minimal segment length (mm), mean ± SD (range)				
1	45.3 ± 16.8 (17.0 – 84.7)	32.0	52.6 ± 12.5 (34.0 – 60.2)	0.261
2	36.7 ± 14.3 (16.0 – 64.8)	37.0 ± 14.7 (28.4 – 59.0)	40.3 ± 16.8 (22.7 ± 73.0)	0.926
3	34.5 ± 14.2 (16.7 – 71.3)	27.1 ± 6.4 (25.8 – 29.5)	–	0.650
Maximal segment length (mm), mean ± SD (range)				
1	53.7 ± 16.4 (29.0 – 91.5)	52	62.2 ± 12.1 (45.0 – 71.5)	0.464
2	49.4 ± 16.1 (20.0 – 80.2)	45.1 ± 14.7 (32.9 – 64.5)	47.2 ± 16.1 (32.3 ± 79.3)	0.809
3	43.1 ± 13.5 (27.4 – 89.5)	36.8 ± 2.1 (29.9 – 42.4)	–	0.508
Length of TTF, mean ± SD (n^‡^)	31.3 ± 12.2 (55)	32.3 ± 11.7 (4)	40.1 ± 14.9 (8)	^‡^0.034

BMI, body mass index; FFF, free fibula flap; PB, periosteal branch; SCP, septo-cutaneous perforator; SD, standard deviation; TTF, truncus tibiofibularis; WHD, wound healing disorder. *Significant difference was only found between flap success and partial flap failure group. ^‡^TTF was only assessed in type I-A branching pattern.

The donor site was in nearly two-thirds of the cases (63.9 %) the right leg, and a minimal distance to the distal tip of the fibular of more than 70 mm was planned in 91.7 % of our cases to preserve ankle stability. TFF has not been observed when tri-segmental jaw reconstruction has been performed.

No significant difference (p = 0.431) was found for the length of TTF concerning flap outcome (FS: 31.3 ± 12.2 mm vs. TFF: 40.1 ± 14.9 mm).

Only when composite flaps were used, wound healing disorders of the donor site were registered. Harvesting defects were standardized covered with a meshed split-thickness skin graft (0.4 mm). The proportion of significant wound healing disorders (WHD) was almost twice as high as that of minor WHD in the FS-group (33.9 % vs. 18.6 %). In the TFF-group, this proportion quadrupled and must be viewed critically due to the small number of cases. No WHD was observed summarized in half of the patients in all groups (**Table 4**).

**Table 4:** Wound healing disorders of the donor site.

*n* = 72	Flap success	Partial flap failure	Total flap failure	*p*-value
	59 (82.0%)	4 (5.5%)	9 (12.5%)	
Composite flap, n (%)				
None	20 (33.9)	1 (25.0)	2 (22.2)	
Minor WHD	11 (18.6)	0	1 (11.1)	
Major WHD	20 (33.9)	2 (50.0)	4 (44.4)	
Non-composite flap				
None	8 (13.6)	1 (25.0)	2 (22.2)	0.523

**Figure 6:**
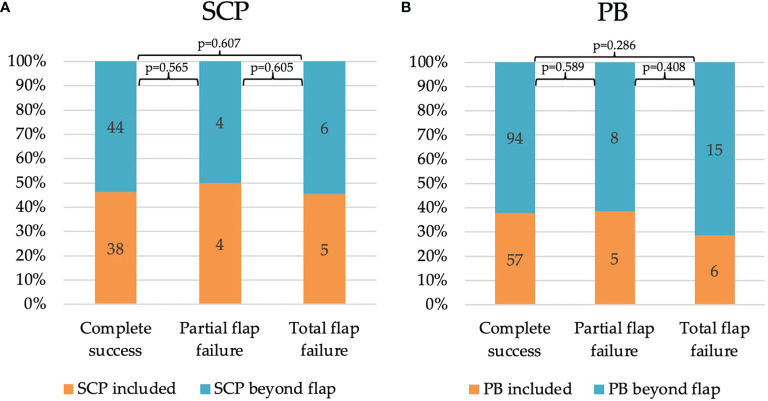
Impact of relative distribution of **(A)** SCP and **(B)** PB concerning FFF outcome: Complete success, *n* = 59; Partial flap failure, *n* = 4; Total flap failure, *n* = 9. An absolute number of recorded vessel types are noted in the bars. Annotation: All possible SCP were recorded without consideration of including a skin paddle (composite FFF type). FFF, free fibula flap; PB, periosteal branch; SCP, septo-cutaneous perforator.

The total number of in FFF included SCP and PB of the FA were analyzed in relation to the found vessels beyond the flap and classified concerning flap outcome (**Figure 6**). No significant differences were observed for different flap outcomes and the number of included SCP (CS 46.3 % vs. PFF 50.0 %, TFF 45.4 %) and PB (CS 37.7 % vs. PFF 38.5 %, TFF 28.6 %).

Further, the number of every single segment of a mono- and poly-segmental reconstruction which was supplied by at least one PB (**Table 5**), and analog for SCP (only for composite flaps, n = 61) (**Table 6**) was assessed. Based on the number of prepared segments, at least one PB supplied 46.7% in the mono-, 40.4% in the bi-, and 31.5 % in the tri-segmental group. Overall, this corresponds to 37.2% (45 out of 121) of all successful FFF. For TFF, a relative number of 42.9% of single addressed segments was calculated. The findings were non-significant. In summary, the number of SCP per segment were lower in poly-segmental composite FFF than in mono-segmental composite reconstruction (**Table 6**). These results are without significance.

**Table 5:** Absolute (n) and relative (%) number of fibular segments were addressed by at least one periosteal branch (PB) based on preoperative CTA for VSP.

PB ≥1 per segment (total segments *n* = 143)	Flap success 121 (= 59 FFF)	Partial flap failure 8 (= 4 FFF)	Total flap failure 14 (= 9 FFF)
1 SFFF, n (%)	7 (46.7)	0	2 (50.0)
2 SFFF, n (%)	21 (40.4)	2 (50.0)	4 (40.0)
3 SFFF, n (%)	17 (31.5)	2 (66.7)	–
All, n (%)	45 (37.2)	4 (50.0)	6 (42.9)

**Table 6:** Absolute (n) and relative (%) number of fibula segments of composite FFF, which were addressed by at least one septo-cutaneous perforator (SCP) based on preoperative CTA for VSP.

SCP ≥1 per segment (total segments *n* = 126)	Flap success	Partial flap failure	Total flap failure
	109 (= 51 FFF)	6 (= 3 FFF)	11 (= 7 FFF)
1 SFFF, n (%)	4 (36.4)	0	2 (66.7)
2 SFFF, n (%)	13 (29.5)	1 (50.0)	3 (37.5)
3 SFFF, n (%)	12 (22.2)	1 (33.3)	–
All, n (%)	29 (26.6)	2 (33.3)	5 (45.4)

**Figure 7:**
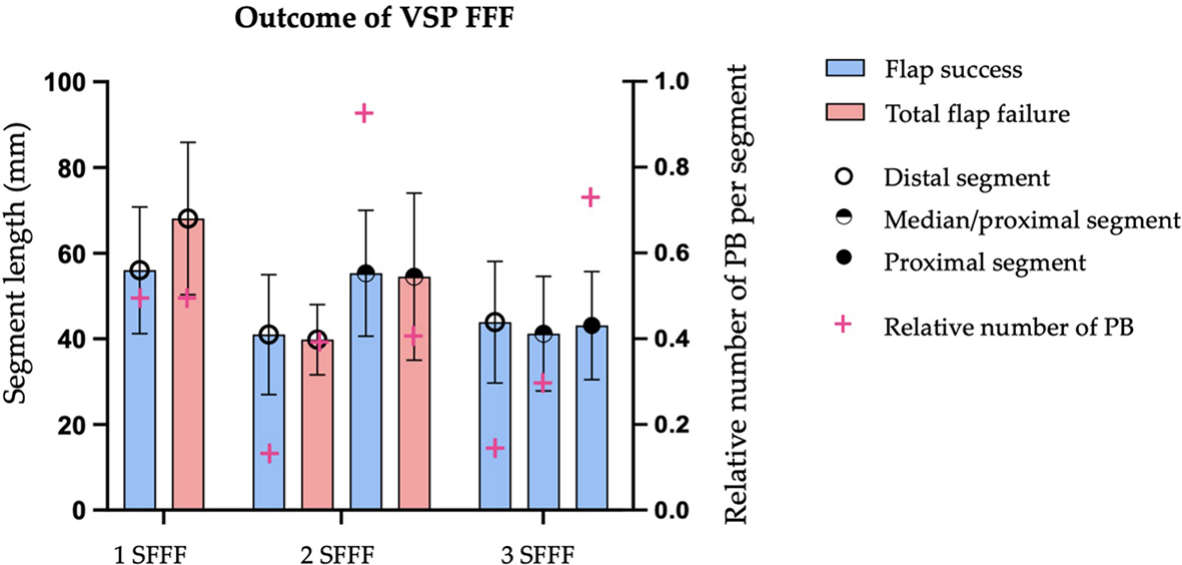
Impact of FFF outcome concerning length of fibular bone segments of mono- (1 SFFF), bi- (2 SFFF) or tri-segmental (3 SFFF) flap for achieving jaw reconstruction (left y-axis). The relative number of periosteal branches (PB) per segment (right y-axis) was calculated and superimposed (magenta cross). 1 SFFF flap success: *n* = 16 vs. total flap failure: *n* = 4; 2 SFFF flap success: *n* = 28 vs. *n* = 5; 3 SFFF flap success: *n* = 19 vs. *n* = 0; Amount of observed PB in region of transplanted fibular bone segments *n* = 66 based on the preoperative CTA.

Minimal and maximal segment length of each virtually shaped FFF segment was assessed and categorized concerning flap outcome. With an increasing number of used FFF segments for reconstruction, the mean segment length decreases (**Table 3**). With the same number of used segments, no statistically significant differences could be found. In detail, the shortest segment length was found in mean with ≥ 34.5 ± 14.2 mm for successful tri-segmental reconstructions, with ≥ 27.1 ± 6.4 mm for partial flap failure in tri-segmental reconstructions, and with ≥ 40.3 ± 16.8 mm for total flap failure in bi-segmental reconstructions. The length of each fibula flap segment was non-significant different in mono- (p = 0.194) and bi-segmental (p = 0.752) reconstructions concerning flap success. In poly-segmental jaw reconstructions, the proximal FFF segments (proximal in bi- and proximal > medial in tri-segmental reconstruction), a higher rate of PB per flap segment was assessed than in the distal segments (**Figure 7**).

## Discussion

Despite advances in the planning of free flaps, improvements of microsurgical techniques (54), and flap monitoring (55–57), the result of surgical reconstruction is still threatened by perfusion disorders of macro- and microcirculation. Flap loss severely disturbs patients’ quality of life and increases the risk of further surgical procedures. Intensive preoperative assessment and imaging evaluation are necessary to decrease peri- and postoperative complications and increase flap success (58–63). CTA has been shown as a sensitive and specific method for microsurgical free flap (21, 64–66) and perforator flap harvesting in reconstructive surgery (67–75).

Over 43 % (n = 31) of the included study subjects were classified at least ASA-score 3. PFF occurred in 2 cases (6.5 %) and TFF in 4 cases (12.9 %). On the other hand, in the ASA-score 1 and 2 groups (n = 41), we documented n = 2 PFF (4.9 %) and n = 5 (12.2 %) TFF. Despite the presence of comorbidities, we did not observe an increase in complications and flap loss. These results are comparable to the literature reported by other study groups (76–78).

### How do Infra-Popliteal Branching Pattern and Fibular Artery Vascular Anomalies (Stenoses) Affect the Outcome of Flap Surgery?

Evaluation of the donor site vascular architecture revealed that type I-A was found in 93.1 % (non-donor site: 84.7 %) according to the classification by Kim et al. (51). Two donor site vascular systems were classified as type I-B, and one case was assigned to categories I-C to II-B. Four legs of the non-donor site showed dominant FA variants (III-A: n = 2; III-B: n = 2). The foot’s blood supply is then shared between FA and non-hypoplastic ATA or PTA in type III-A and B, and FA is enlarged as a result (79–81). It was previously estimated that 5.2 % of limbs have dominant FAs (66). The study sample presented either on the donor or non-donor site none peroneal artery magna (type III-C), in which FA supplies blood to the lower leg and foot.

Overall, the distribution of the recorded branching variants of the popliteal artery is comparable to previous published data (82). However, it is imperative to identify this particular singular vasculature before FFF harvesting to prevent critical limb and foot ischemia (63, 83, 84). The investigation revealed that all but one of the PFF and TFF cases could be assigned to type I-A and I-B branching patterns. In accordance with the literature, type I-A is the most common branching pattern. Successful flap transfers occurred in types I-B, I-C, II-A, and II-B (each n = 1).

9.7 % of cases with vascular stenoses (n = 14) were identified in the sample, and from these, 11 were localized in the FA. There were five stenoses in the distal course of the FA run-off at the donor site, and two of these were associated with TFF. On the other hand, three cases of FA stenoses did not impact flap success. Remarkably, the majority of the recorded stenoses were located in FA. Other studies suggest the FA is not as severely affected by the peripheral arterial occlusive disease (PAOD) as the tibial arteries (60, 85, 86). Despite vascular calcifications impacting the flap vascular pedicle, successful microvascular FFF has been described, with a 0 % complete flap failure rate and a 7 % partial flap failure rate (87). Preoperative optimizing of leg perfusion by endovascular interventions has also been reported as a therapeutic option in possible critical limb perfusion (88).

Further study findings revealed significant differences concerning the length of TTF in the flap failure group compared to flap success and were assessed with an extended length of 40.1 ± 14.9 mm (p = 0.034). A more prolonged TTF implicates a decreasing length of the FA and, therefore, the entire vascular pedicle of the FFF. While short pedicle length can aggravate microsurgical anastomosis (89), a long pedicle is endangered for kinking and twisting with critical blood flow of the vascular axis (90). Published literature hypothesizes a relation between length and course of TTF and high body mass. This condition may contribute to enlarged and curved/twisted TTF, promoting local atherosclerosis and impeding microsurgery (91).

Summarized, the infra-popliteal branching pattern types I-A to II-B did not affect the flap surgery outcome in the present study. Furthermore, despite recorded vascular stenoses of the FA, flap success was observed in more than the half of those cases.

### How Does the Distribution of CTA-Based Detected PB and SCP Influence the Surgical Result of Mono- and Poly-Segmental Jaw Reconstructions With Partial or Total Flap Loss?

The results of this study show that a PB and SCP (musculo-fascio-periosteal perforators) could not be visualized in every virtually planned and transplanted segment in the preoperatively performed lower limb CTA scan. Nevertheless, mono- and poly-segmental reconstructions were successful when no PB was found in CTA evaluation and failed, although PB (and SCP) were verifiable.

When matching harvested segments with detected PB, 38.5 % of all virtual planned segments (n = 143 in 72 patients) were congruent to one or more PB localization. If the segments which at least one PB distinctively supplied are assigned to the defined flap outcome groups, it was found that the FS group has the lowest rate with 37.2 %, the PFF group has the highest rate with 50.0 %, and the TFF group is between both with 42.9 % (**Table 5**). Therefore, the rate of in CTA detected PB found per segment did not provide information concerning expected flap success. The explored distribution patterns reflect PB and SCP clustering and confirm the high variability of the localization and course. It is noticeable that in poly-segmental reconstructions, the probability of observing a PB in the CTA increases in the more proximal segment. Previous investigations on the study sample revealed a bimodal distribution pattern for PB and three peaks for SCP in performed CTA for VSP (48). These patterns of distribution are similar to the results of other studies (17, 20, 92).

Investigations of CT-scans in fresh frozen cadaver lower limbs showed in mean 12.8 periosteal branches of the fibular artery with a mean intersegmental distance of 1.36 cm between them, and at least one branch in 65.1% in 1.0 cm segments, and up to 94% of the 2.0 cm segments (49). Their radiological findings of the detected periosteal branches (49) support the measurements of fibular segments perfusion in cadavers (93), but differ from our conclusions presented. The previous analysis of the study sample has shown that in 10.8 %, one PB was found in a 1.0 cm fibula section in our defined region of interest between the origin of the FA and a plane 5 cm above the distal tip of the fibula section. The likelihood increases in 2.0 cm segment up to 21.1 % and in 3.0 cm segment length to 29.2 %, having included at least one PB (48). Further, there is no difference regarding the density of periosteal and musculo-periosteal vessels in the long and short fibula segments. Existing collaterals between the superficial, periosteal, and the internal endoperiosteal system, were not able to compensate for the work of the non-functional vessels if the segment length was too short. However, this is more likely to occur if the segments are longer (93). Larger segments and fewer osteotomies were associated with higher perfusion (94). Battaglia et al. reported a series of 20 patients in matching in CTA images identified perforators with the intraoperative perforator location while FFF harvesting (65). An average distance between CTA perforator positions and intraoperative perforator positions of 1 mm (range 0 to 2 mm) was assessed. They concluded that preoperative CTA evaluation to investigate lower-extremity vascular patterns for patients undergoing composite FFF is a valuable approach for reducing VSP complications due to variable vascular anatomy. Still, more follow-up studies are needed to assess this modern technique’s long-term outcomes and benefits (65). Ettinger et al. report that further development of CTA imaging protocols and existing VSP workflows is necessary to be optimized to allow faster and more accurate preoperative modeling of cutaneous perforator anatomy for consideration in VSP of reconstructions (64). These authors point out also, that CTA for VSP allows taking the position of perforators into account when planning poly-segmental reconstruction and skin paddle (64). A previous study found that CTA could detect the size, course, and penetration pattern of all perforators with a diameter more than 0.3 mm (21). Recent investigation on the study population confirmed these statements (48). However, it can be assumed that the discrepancy in anatomical findings is based on the quality of the CTA scans. Several other factors influence CTA scan accuracy, including the timing, dosage, and coordination of the contrast bolus with the sequence of images (95).

Overall, the rate of CTA detected PB per segment did not indicate flap success. Mono- and poly-segmental reconstructions were successful when no PB (and SCP) were found in the CTA evaluation and even were unsuccessful when PB (and SCP) were recorded.

### Does the Observed Distribution of PB and SCP Impact Wound Healing of the Donor Site?

WHD of the donor site were recorded only in the composite flap group, and the proportion of WHD was less high in the TFF-group (55.5 %) than in the FS-group (52.5 %). The differences should be viewed critically according to the small number of cases. A separation between minor WHD (small wound area and local therapy) and major WHD (large wound, exposed tendon, and need of surgical therapy with debridement, new skin grafting) had been done and showed, that major WHD (33.9%) had been recorded near to twice than minor WHD (18.6%) in the FS-group. In summary, more than 52.8 % of the entire study sample WHD were found. Published literature shows complication rates from 0% to 33% (62, 96, 97). In the present study, donor site defect of composite FFF were covered in all cases with a meshed split-thickness skin graft, and every (sub-)total graft loss was counted and defined as WHD. Primarily wound closure was only performed after non-composite FFF harvesting, and wound healing disorder was not found in this group.

According to SCP per segment matching rate, only composite flaps were evaluated. A total number of n = 126 segments in 64 patients has shown that overall, 28.6 % of all virtual planned segments were congruent to one or more SCP localization. However, this finding does not allow providing information concerning wound healing disorder. On the one hand, the authors believe that the size of the skin paddle and the donor site defect, and the patient’s general condition with comorbidities play a decisive role in wound healing. Heavy tobacco use was found to have as a risk factor for wound impairment (97).

The problem of WHD as a donor site morbidity has been known in the literature for a long time. Up to now, closure of the donor site is controversial and ranges from primarily closure, open wound healing, split-thickness skin graft, full skin graft, free flap (96, 98). Open healing of the fibular donor site and meshing of the surrounded tissue has been reported as a modification to decrease the wound area and avoid the morbidity associated with graft and resulted in a good cosmetic outcome (99). The use of vacuum-assisted closure allows patients to be mobilized sooner, assures greater graft acceptance, and reduces healing time up to 50% (100).

Up to now, information about the number and course of PB and SCP has not been of interest in our entire virtual planning process. Designing the composite flap and especially the skin paddle’s dimension depends on the defect size and visible SCP in the posterior intermuscular septum. From our clinical experience, we agree with others that handheld Doppler sonography examination is often unsuitable in general anesthesia to identify SCP reliably. Identifying tiny perforators and distinguishing between superficial muscular perforators and cutaneous perforators is difficult (101). Islam et al. discovered that real-time, color-flow Doppler ultrasonography was beneficial in the planning and harvesting free perforator flaps and suggested that it be used more widely than traditional hand-held Doppler equipment (102). We prefer the direct assessment and visualization of the SCP during dissection (**Figure 3**).

The distribution of in preoperative CTA detected PB and SCP per segment was not associated with the rate of wound healing disorders of the donor site after composite flap harvesting.

### Limitations of the Study

There are some limitations in this retrospective study. Only patients who underwent the following FFF procedure were included in the investigation. Patients who were not suitable for FFF after CTA scan were not included, and the number of cases remains unclear. The investigated study population consisted a mixture of malignant and benign diseases which give an inhomogeneity to the study subjects. Another limitation is that multiple surgeons were involved in the treatment of the study population. Three different surgeons were involved in FFF harvesting over the entire study period. Evaluated CTA scans were not performed under experimental, controlled conditions. Instead, they were run as routine clinical imaging which reported by different radiologists.

Furthermore, as concluded in a previous study, the in CTA observed number of PBs and SCPs is substantially less than accurate as the anatomical findings (48). Therefore, the number of small vessels could be underestimated. Further studies using better developed volume visualization software to improve the illustration of small vessels are necessary as a future research step in this topic.

## Conclusion

Preoperatively CTA for VSP of free fibula flap (FFF) is suitable for vascular mapping of the infra-popliteal vascular system and smaller vessels. Despite recorded stenoses of fibular artery in five cases, FFF was in 60% successful.

Correlation between higher rates of PB, SCP and the flap success could not be statistically proved in study sample. We conclude, that preoperative PB and SCP mapping based on routine CTA imaging is not suitable for prediction of flap outcome.

## Data Availability Statement

The raw data supporting the conclusions of this article will be made available by the authors, without undue reservation.

## Ethics Statement

The study was approved by the local Ethics Committee of Justus-Liebig University Giessen (AZ33/20, approval 25.5.2020). Written informed consent for participation was not required for this study in accordance with the national legislation and the institutional requirements.

## Author Contributions

Conceptualization, MK and SA. Data curation, DS and SB. Formal analysis, MK and PS. Funding acquisition, H-PH. Investigation, MK and AB. Methodology, MK, AB, CA, and FR. Supervision, H-PH and SA. Validation, SA. Visualization, CB. Writing – original draft, MK. Writing – review & editing, AB, CB, CA, FR, DS, SB, PS, H-PH, and SA. All authors have read and agreed to the published version of the manuscript.

## Conflict of Interest

The authors declare that the research was conducted in the absence of any commercial or financial relationships that could be construed as a potential conflict of interest.

## Publisher’s Note

All claims expressed in this article are solely those of the authors and do not necessarily represent those of their affiliated organizations, or those of the publisher, the editors and the reviewers. Any product that may be evaluated in this article, or claim that may be made by its manufacturer, is not guaranteed or endorsed by the publisher.
